# Complete genome sequence of sorbitol-fermenting, *β*-glucuronidase-positive, non-motile *E*. *coli* O157:H7 isolated from clinical stool specimen

**DOI:** 10.1128/mra.00035-26

**Published:** 2026-03-16

**Authors:** Haejin Hwang, Melanie Orth, Matthew Plumb, Jisun Haan

**Affiliations:** 1 Infectious Disease Laboratory Section, Minnesota Department of Health, Public Health Laboratory 11055, Saint Paul, Minnesota, USA; Wellesley College, Wellesley, Massachusetts, USA

**Keywords:** O157, sorbitol positive, *E.coli* O157

## Abstract

We report the complete genome of a sorbitol-fermenting, *β*-glucuronidase-positive, non-motile *Escherichia coli* O157:H7 isolated from a human stool sample. The genome consisted of a 5,487,050-bp circular chromosome and a 79,741-bp F-like plasmid.

## ANNOUNCEMENT

Shiga toxin-producing *Escherichia coli* (STEC) O157:H7 is a major foodborne pathogen that can cause hemorrhagic colitis and life-threatening hemolytic uremic syndrome (1). The inability of *E. coli* O157:H7 to ferment sorbitol or produce *β*-glucuronidase distinguishes it from other non-O157 STEC strains (2, 3). However, Shiga toxin-producing, sorbitol-fermenting *E. coli* O157:H7 strains have been documented (4), including a 2011 outbreak in France (5).

**Fig 1:**
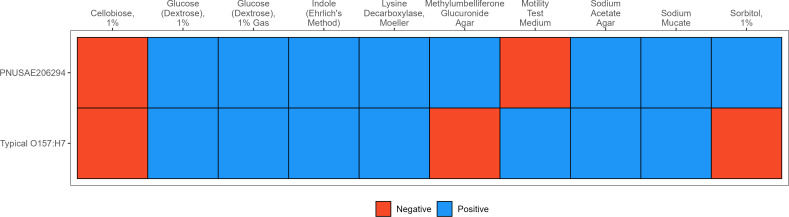
Binary heatmap comparing biochemical profiles of a sorbitol-positive, β-glucuronidase-positive, non-motile *Escherichia coli* O157:H7 strain (PNUSAE206294) and a typical *E. coli* O157:H7 strain. Each cell indicates a positive (blue) or negative (red) result for the corresponding biochemical test shown on the *x*-axis**.** Sorbitol-positive colony was picked from the SMAC plate and sub-cultured on trypticase soy agar with 5% sheep blood at 36°C for 18–24 h. The isolate was inoculated into the biochemicals listed in Figure 1 and incubated at 36°C for 48 h. All biochemicals were prepared in-house, except the motility test medium (VWR).

Here, we report the complete genome of a sorbitol-fermenting, *β*-glucuronidase-positive, non-motile *E. coli* O157:H7 isolated from a clinical stool specimen. The Minnesota Department of Health—Public Health Laboratory (MDH-PHL) received a stool specimen reported as O157-positive via the BioFire FilmArray Gastrointestinal Panel. Initial culture on sorbitol-MacConkey (SMAC) agar plate and sorbitol-MacConkey agar with cefixime and tellurite (CT-SMAC) plate at 36°C for 24 h showed sorbitol-positive colonies. Immunomagnetic separation (IMS) using Dynabeads (Thermo Fisher) was performed on the stool aliquot to isolate O157 colonies, which were cultured on CT-SMAC plates. The IMS culture also resulted in sorbitol-positive colonies, suggesting potential non-O157 *E. coli* isolates. The Shiga toxin genes (*stx1* and *stx2*) and the intimin gene (*eae*) were detected by real-time multiplex PCR from the SMAC plate (6). An isolated colony was sub-cultured and underwent biochemical testing for confirmation (7, 8). The results of biochemical testing can be found in Fig. 1.

Genomic DNA was extracted from a 1 µL loopful of an overnight culture on trypticase soy agar using the QIAamp DNA Mini QIAcube Kit on a QIAcube following the manufacturer’s instructions. The same DNA extract was used to prepare Illumina and Oxford Nanopore Technologies (ONT) sequencing libraries. The Illumina sequencing library was prepared with the Illumina DNA Prep 96 Sample Kit and sequenced on an Illumina NextSeq 2000 using NextSeq 1000/2000 P1 300 cycles. The ONT library was constructed with a rapid barcoding kit (SQK-RBK114.24) and sequenced on a GridION MK1 using an R10.4.1 flow cell. Nanopore reads were basecalled using Dorado v.7.6.7 with super-accuracy base-calling mode.

Default parameters were used unless otherwise noted. Nanopore reads were filtered with Filtlong v.0.2.1 (9), and Illumina reads with fastp v.1.0.0 (10). Hybrid assembly was performed with Unicycler v.0.5.1 (11). The overlap was identified and trimmed automatically using assembly graph structure and long-read bridging. The chromosome and plasmid were automatically rotated by Unicycler. Genome annotation was performed with PGAP v.2024-07-18.build7555 (12). Assembly completeness was checked with BUSCO v.5.8.2 (13) by counting the set of single-copy marker genes from the Enterobacteriaceae orthologs database (Enterobacteriaceae_odb12) found in the assembly. Assembly metrics were checked with QUAST v.5.2.0 (14). The serotype was determined using ECTyper ver.2.0.0 (15). ABRicate v.1.0.1 (16) was used to detect plasmids, Shiga toxin, and other virulence genes against the PlasmidFinder (17) and Virulence Factor Database (VFDB) (18). The *repE* gene from the annotated plasmid was BLASTed against the core_nt database (19, 20).

Sequencing and annotation (Table 1) showed an assembled genome of 5,566,791 bp, with a circular chromosome (5,487,050 bp), an F-like plasmid (79,741 bp), and a guanine or cytosine (G + C) content of 50.5%. The BUSCO genome completeness score was 99.8%. The chromosome contained 5,232 genes, and the plasmid contained 83 genes. The predicted serotype based on whole-genome sequencing was O157:H7. The Shiga toxin genes *stx2A* and *stx2B* and the intimin gene were detected. The plasmid *repE* gene matched *E. coli* O157:H7 strain 493/89 plasmid p493-89-1 (GenBank accession: CP038413.1) with 100% identity and zero gaps.

**TABLE 1:** Summary of sequence data and genome features

	Metric	Value
Illumina	Total number of reads sequenced	2,003,320
	N50 (no. of bases)	150
	Genome coverage	54
Oxford Nanopore	Total number of reads sequenced	101,680
	N50 (no. of bases)	12,421
	Genome coverage	84
Assembled genome	Number of contigs	2
	Total genome size (no. of bases)	5,566,791
	Size of chromosome (no. of bases)	5,487,050
	Size of plasmid (no. of bases)	79,741
	G + C content (%)	50.5
	Genome completeness by BUSCO (%)	99.8
Chromosome	Number of genes	5,227
	Number of CDSs	5,098
	Number of tRNAs	101
	Number of rRNAs	22
Plasmid	Number of genes	83

## Data Availability

The sequence reads and assembled genome have been deposited in SRA and GenBank databases under accession numbers SRR32632538 (Illumina), SRR35172242 (Oxford Nanopore), and ABYFJL020000000 (assembled genome and plasmid) (BioProject: PRJNA218110; BioSample: SAMN47290339; strain identifier: PNUSAE206294).

## References

[1] Lim JY, Yoon J, Hovde CJ. 2010. A brief overview of Escherichia coli O157:H7 and its plasmid O157. J Microbiol Biotechnol 20:5–14. doi:10.4014/jmb.0908.08007 20134227 PMC3645889

[2] March SB, Ratnam S. 1986. Sorbitol-macconkey medium for detection of Escherichia coli O157:H7 associated with hemorrhagic colitis. J Clin Microbiol 23:869–872. doi:10.1128/jcm.23.5.869-872.1986 3519658 PMC268739

[3] Thompson JS, Hodge DS, Borczyk AA. 1990. Rapid biochemical test to identify verocytotoxin-positive strains of Escherichia coli serotype O157. J Clin Microbiol 28:2165–2168. doi:10.1128/jcm.28.10.2165-2168.1990 2229338 PMC268139

[4] Rosser T, Dransfield T, Allison L, Hanson M, Holden N, Evans J, Naylor S, La Ragione R, Low JC, Gally DL. 2008. Pathogenic potential of emergent sorbitol-fermenting Escherichia coli O157:NM. Infect Immun 76:5598–5607. doi:10.1128/IAI.01180-08 18852247 PMC2583558

[5] King LA, Loukiadis E, Mariani-Kurkdjian P, Haeghebaert S, Weill F-X, Baliere C, Ganet S, Gouali M, Vaillant V, Pihier N, Callon H, Novo R, Gaillot O, Thevenot-Sergentet D, Bingen E, Chaud P, de Valk H. 2014. Foodborne transmission of sorbitol-fermenting Escherichia coli O157:[H7] via ground beef: an outbreak in northern France, 2011. Clin Microbiol Infect 20:O1136–44. doi:10.1111/1469-0691.12736 24962059

[6] Pavlovic M, Huber I, Skala H, Konrad R, Schmidt H, Sing A, Busch U. 2010. Development of a multiplex real-time polymerase chain reaction for simultaneous detection of enterohemorrhagic Escherichia coli and enteropathogenic Escherichia coli strains. Foodborne Pathog Dis 7:801–808. doi:10.1089/fpd.2009.0457 20156086

[7] Atkinson R, Besser J, Bopp C, Carlson C, Crandall C, George K, Gerner-Smidt P, Gladbach S, Gould L, Hartley C, Maguire H, Monson T, Rutledge D, Shea S, Somsel P, Strockbine N. 2012. Guidance for public health laboratories: isolation and characterization of shiga toxin-producing Escherichia coli (STEC) from clinical specimens. Available from: https://stacks.cdc.gov/view/cdc/21592

[8] Carroll K, et al.. 2023. Manual of clinical microbiology. ASM Press, American Society for Microbiology.

[9] Wick R, Menzel P. 2019. Filtlong: quality filtering tool for long reads. Github

[10] Chen S. 2025. Fastp 1.0: an ultra‐fast all‐round tool for FASTQ data quality control and preprocessing. iMeta 4. doi:10.1002/imt2.70078 PMC12527978 41112039

[11] Wick RR, Judd LM, Gorrie CL, Holt KE. 2017. Unicycler: resolving bacterial genome assemblies from short and long sequencing reads. PLOS Comput Biol 13:e1005595. doi:10.1371/journal.pcbi.1005595 28594827 PMC5481147

[12] Tatusova T, DiCuccio M, Badretdin A, Chetvernin V, Nawrocki EP, Zaslavsky L, Lomsadze A, Pruitt KD, Borodovsky M, Ostell J. 2016. NCBI prokaryotic genome annotation pipeline. Nucleic Acids Res 44:6614–6624. doi:10.1093/nar/gkw569 27342282 PMC5001611

[13] Manni M, Berkeley MR, Seppey M, Simão FA, Zdobnov EM. 2021. BUSCO update: novel and streamlined workflows along with broader and deeper phylogenetic coverage for scoring of eukaryotic, prokaryotic, and viral genomes. Mol Biol Evol 38:4647–4654. doi:10.1093/molbev/msab199 34320186 PMC8476166

[14] Gurevich A, Saveliev V, Vyahhi N, Tesler G. 2013. QUAST: quality assessment tool for genome assemblies. Bioinformatics 29:1072–1075. doi:10.1093/bioinformatics/btt086 23422339 PMC3624806

[15] Bessonov K, Laing C, Robertson J, Yong I, Ziebell K, Gannon VPJ, Nichani A, Arya G, Nash JHE, Christianson S. 2021. ECTyper: in silico Escherichia coli serotype and species prediction from raw and assembled whole-genome sequence data. Microb Genom 7. doi:10.1099/mgen.0.000728 PMC8767331 34860150

[16] Seemann T. 2020. ABRicate. https://github.com/tseemann/abricate.

[17] Carattoli A, Zankari E, García-Fernández A, Voldby Larsen M, Lund O, Villa L, Møller Aarestrup F, Hasman H. 2014. In silico detection and typing of plasmids using PlasmidFinder and plasmid multilocus sequence typing. Antimicrob Agents Chemother 58:3895–3903. doi:10.1128/AAC.02412-14 24777092 PMC4068535

[18] Chen L, Zheng D, Liu B, Yang J, Jin Q. 2016. VFDB 2016: hierarchical and refined dataset for big data analysis—10 years on. Nucleic Acids Res 44:D694–D697. doi:10.1093/nar/gkv1239 26578559 PMC4702877

[19] Zhang Z, Schwartz S, Wagner L, Miller W. 2000. A greedy algorithm for aligning DNA sequences. J Comput Biol 7:203–214. doi:10.1089/10665270050081478 10890397

[20] Morgulis A, Coulouris G, Raytselis Y, Madden TL, Agarwala R, Schäffer AA. 2008. Database indexing for production MegaBLAST searches. Bioinformatics 24:1757–1764. doi:10.1093/bioinformatics/btn322 18567917 PMC2696921

